# Left atrial remodeling and voltage-guided ablation outcome in obese patients with persistent atrial fibrillation

**DOI:** 10.3389/fcvm.2024.1362903

**Published:** 2024-04-02

**Authors:** Halim Marzak, Simon Fitouchi, Aïssam Labani, Justine Hammann, Romain Ringele, Mohamad Kanso, Thomas Cardi, Alexandre Schatz, Patrick Ohlmann, Olivier Morel, Laurence Jesel

**Affiliations:** ^1^ Division of Cardiovascular Medicine, Nouvel Hôpital Civil, Strasbourg University Hospital, Strasbourg, France; ^2^ Department of Radiology, Nouvel Hôpital Civil, Strasbourg University Hospital, Strasbourg, France; ^3^ INSERM (French National Institute of Health and Medical Research), UMR 1260, Regenerative Nanomedicine, FMTS, Strasbourg, France

**Keywords:** atrial fibrillation, catheter ablation, obesity, low-voltage zones, bipolar voltage map, atrial remodeling, epicardial adipose tissue

## Abstract

**Background:**

Obesity is a risk factor for atrial fibrillation (AF). Data regarding left atrial (LA) remodeling in obese patients are scarce. Whether obesity favors AF recurrence after catheter ablation (CA) is still controversial. We assessed the distribution of epicardial atrial fat on computed tomography (CT), LA bipolar voltage, low-voltage zone (LVZ) extent, and the outcome of voltage-guided ablation of persistent AF in obese and non-obese patients.

**Methods:**

A total of 139 patients with persistent AF undergoing a first voltage-guided ablation were enrolled and divided into two groups: 74 were non-obese and 65 were obese. Epicardial adipose tissue (EAT) was assessed on a CT scanner. LA endocardial voltage maps were obtained using a 3D mapping system in sinus rhythm. LVZ was defined as a bipolar peak-to-peak voltage amplitude <0.5 mV.

**Results:**

LA volume, voltage, and EAT amount were similar in the two groups. LVZ was less frequent in obese patients [12 (18.8%) vs. 26 (35.1%), *p* = 0.05], particularly on the anterior wall. The posterior and lateral EATs were correlated with posterior and lateral LVZ extent, respectively, in obese patients. After 36 months of follow-up, the AF-free survival rate was similar. Lateral EAT [odds ratio (OR) 1.21, 95% confidence interval (CI) 1–1.4, *p* = 0.04] and P-wave duration (OR 1.03, 95% CI 1–1.05, *p* = 0.03), but not body mass index (BMI), were predictors of AF recurrence after CA.

**Conclusion:**

In obese patients, LVZ was less marked than in non-obese patients with similar LA volumes, voltage, and EAT amounts. In obese patients, posterior and lateral EATs were correlated with posterior and lateral LVZ extents. Obese patients had a similar and favorable 36-month outcome after AF ablation. BMI was not predictive of AF recurrence.

## Introduction

Obesity is associated with an increased cardiovascular mortality and morbidity (1). Several studies have demonstrated a strong association between obesity and atrial fibrillation (AF) occurrence (2). For every 5 kg/m^2^ increase in body mass index (BMI), the risk of AF could increase from 10% to 30% (3). Obesity has been identified as an independent risk factor for AF (2). A stable weight loss ≥10% in obese patients with AF is associated with a decrease in both AF recurrence and burden, and an improvement in AF symptom severity (4, 5). Recently, epicardial adipose tissue (EAT) extent has been associated with AF incidence and severity (6, 7). The Framingham Heart Study indicated that higher pericardial fat volume was associated with a nearly 40% higher odds of prevalent AF (6). Batal et al. reported that patients with persistent AF had a significantly thicker posterior EAT associated with increased AF burden independent of age, BMI, or left atrial (LA) area (8). Finally, Wong et al. showed that the patients with an extensive EAT had early recurrence after AF catheter ablation (CA) (7).

This EAT, located between the visceral pericardium and epicardium surface, is considered a metabolically active tissue. In a model of adult rat, human epicardial, but not subcutaneous adipose, tissue secretome could induce atrial fibrosis, a local effect mediated by activin A, an adipo-fibrokine (9). In addition, subepicardial fatty infiltration was also observed in patients with AF with abundant EAT on histological sections. Haemers et al. observed that these fatty infiltrates could progressively become fibrosis affecting subepicardial areas of the atrial myocardium (10). Low-voltage zones (LVZ) recorded with LA three-dimensional (3D) mapping systems are a powerful predictor of recurrence after AF ablation (11). In a model of obese sheep, LA enlargement, conduction abnormalities, and fractionated electrograms were associated with a reduced posterior LA endocardial voltage (12). In obese humans, data on electrophysiological remodeling are scarce. Whether obese patients display more EAT and associated LVZ is still discussed. AF recurrence after catheter ablation in obese patients also remains debated.

The purpose of our study was to evaluate the regional epicardial atrial fat distribution and assess the extent of LVZ and LA regional bipolar voltage amplitude in a cohort of obese patients with persistent AF compared to a cohort of non-obese patients. The 36-month outcome of LA voltage-guided substrate ablation in addition to pulmonary vein isolation (PVI) and predictors of AF recurrence after CA were also assessed.

## Methods

### Study population

**Figure 1:**
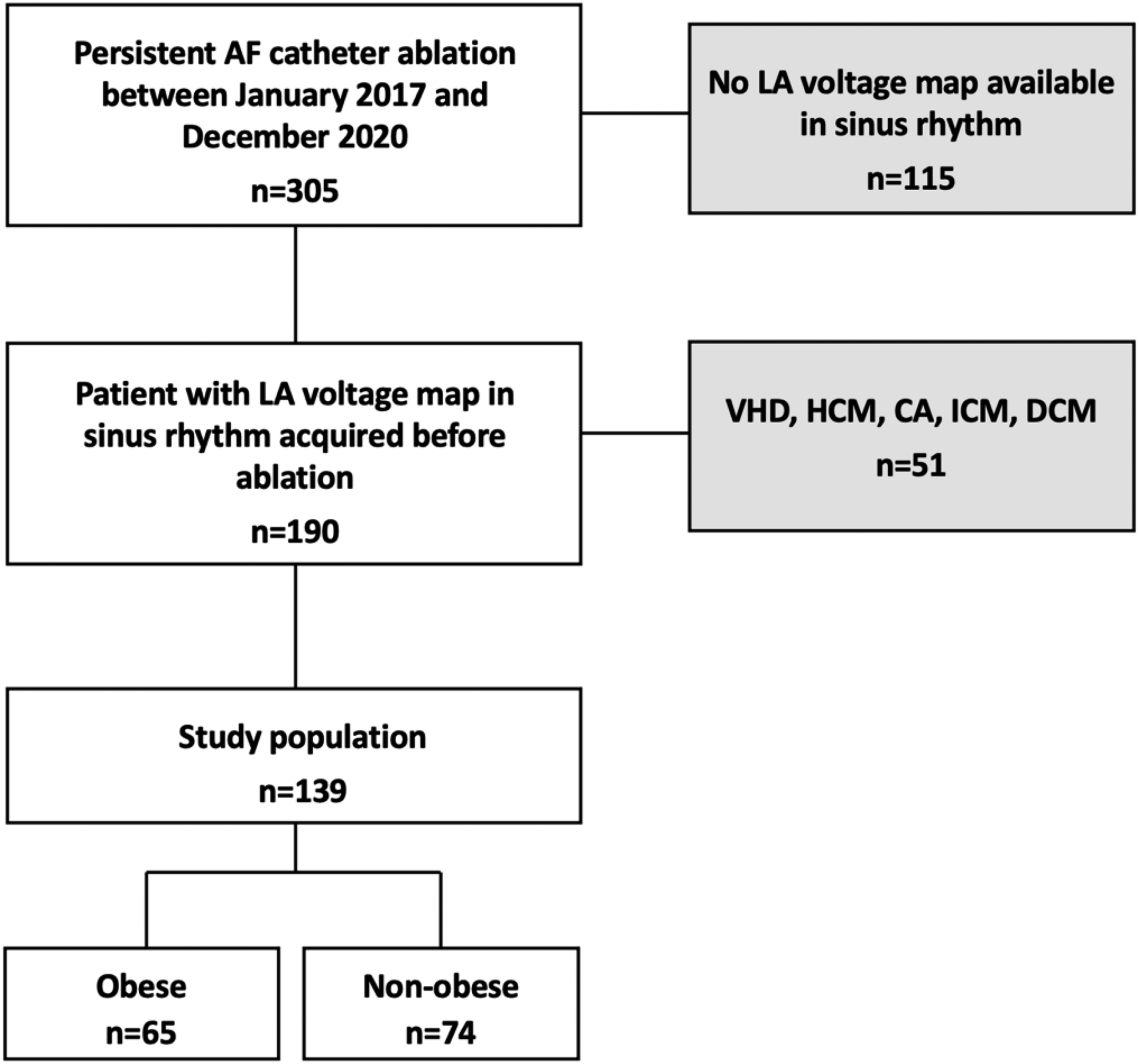
Flow chart of the study. Among 305 patients admitted for initial PAF ablation between November 2017 and December 2020, 139 (45.6%) patients met the inclusion criteria. 115 patients (37.7%) had no LA voltage mapping in sinus rhythm and 51 (16.7%) were excluded from analysis due to structural heart disease. VHD, valvular heart disease; HCM, hypertrophic cardiomyopathy; ICM, ischemic cardiomyopathy; DCM, dilated cardiomyopathy; PAF, persistent atrial fibrillation.

This prospective observational study enrolled 139 consecutive patients presenting for a first persistent AF catheter ablation in our institution. Patients without LA voltage maps obtained in sinus rhythm (SR) and those with structural heart disease were excluded (Figure 1).

Structural heart disease was defined as a previous diagnosis of ischemic heart disease, valve dysfunction (≥moderate), or primary myocardial structural disease including dilated cardiomyopathy and hypertrophic cardiomyopathy. Obesity was defined as BMI ≥30 kg/m^2^. The cohort was divided into two groups: non-obese [with a BMI <30 kg/m^2^ (*n* = 74)] and obese [with a BMI ≥30 kg/m^2^ (*n* = 65)]. Patient demographics and baseline clinical characteristics were collected. The study protocol was approved by the institutional review board of Strasbourg University (CE-2023-113). All patients have given their written informed consent for the ablation and participation in this study. Our obese patients were aware of weight loss to improve the results of AF ablation. Therefore, we would advise them to change their lifestyle (diet and exercise) for weight loss before and after AF ablation.

### Epicardial adipose tissue measurement

Images were acquired using a 320-row CT scanner (Aquilion ONE Genesis; Canon Medical Systems). Image analysis was performed on a dedicated workstation (AW4.6 workstation; GE Healthcare). All CT scanners were reconstructed in three dedicated cardiac planes, centered on a line (using body imaging planes) from the cardiac apex to the middle of the mitral valve.

**Figure 2:**
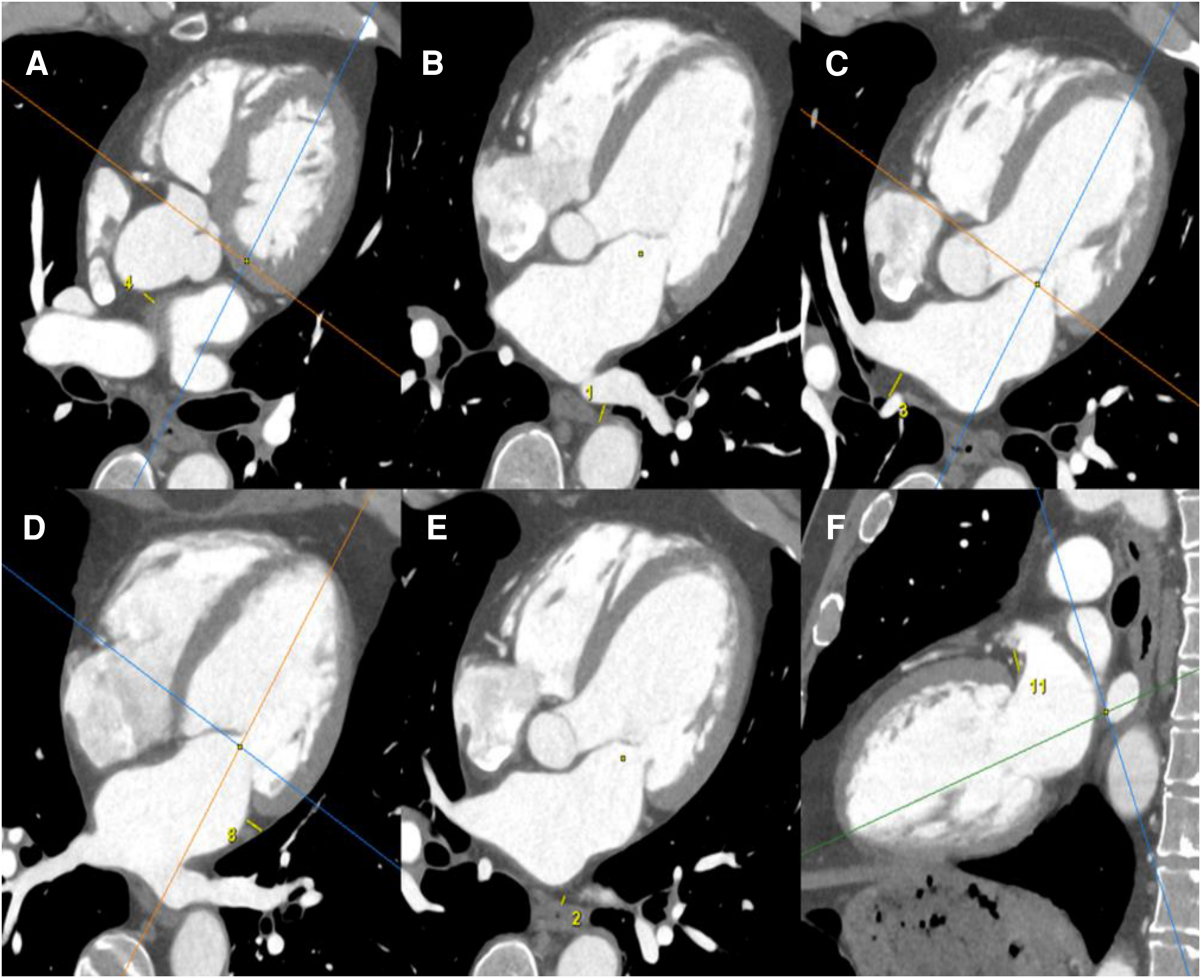
EAT measurements were obtained using a cardiac CT scanner. All CT scanners were reconstructed in three dedicated cardiac planes, centered on a line (using body imaging planes) from the cardiac apex to the middle of the mitral valve: short axis plane: perpendicular to true long axis at the level of mid left ventricle; horizontal long axis plane (four-chamber view): horizontal plane perpendicular to short axis; vertical long axis plane (two-chamber view): vertical plane orthogonal to short axis. EAT was defined as the adipose tissue between the surface of the myocardium and the epicardium. EAT thicknesses were measured by the same radiologist in mm, by manual 2D caliper (small yellow line), as the distance between every LA wall and anatomical landmarks: EAT thickness between anterior LA wall and ascending aorta (**A**); EAT thickness between posterior LA wall and descending thoracic aorta (**B**); EAT thickness between posterior LA wall and right pulmonary hila (**C**); EAT thickness between lateral LA wall and visceral pleura of left lung (**D**); EAT thickness between posterior LA wall and esophagus (**E**); EAT thickness between periauricular lateral LA wall and mitral root (**F**).

EAT was defined as the adipose tissue between the surface of the myocardium and the epicardium. EAT thickness was measured by the same radiologist in millimeters, using a manual 2D caliper, by measuring in the area of maximum thickness the distance between every LA wall and anatomical landmarks, such as the esophagus, for the posterior wall (Figure 2).

### Procedural preparation

Patients were efficiently anticoagulated for more than 3 weeks. The antiarrhythmic drugs (AADs) were interrupted for ≥5 half-lives before catheter ablation. Thus, amiodarone was discontinued 3 weeks before the procedure. Electrophysiological studies and catheter ablation procedures were performed under general anesthesia using a 3D electroanatomical mapping (3D-EAM) system (CARTO 3, Biosense Webster, Diamond Bar, CA, USA, or EnSite Velocity, Abbott, St Paul, MN, USA) and a deflectable decapolar circular mapping catheter (Lasso catheter of variable diameter size (15–25 mm), interelectrode spacing 6 mm (Biosense Webster, Diamond Bar, CA, USA, or a spiral multipolar pulmonary vein catheter, Afocus II, diameter 20 mm, electrode spacing 5 mm, Abbott, St Paul, MN, USA). A transesophageal echocardiography was performed at the beginning of the procedure, to both exclude any thrombi in the left atrial appendage (LAA) and guide the transseptal puncture.

### LA voltage mapping

**Figure 3:**
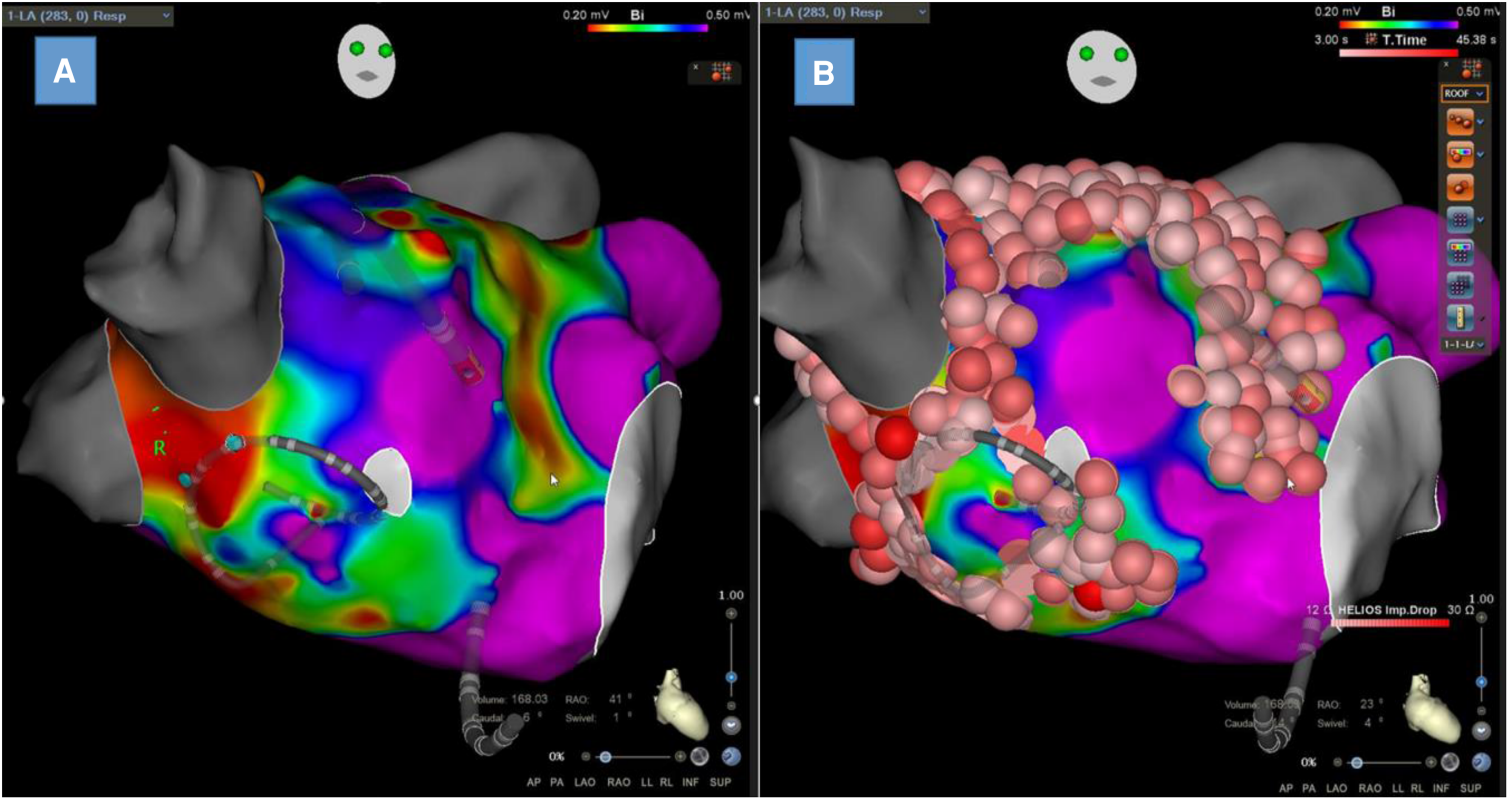
Left atrial bipolar voltage map obtained in sinus rhythm with Lasso™ NAV from Biosense Webster with visualization of low-voltage zones at the anterior wall and septum (**A**) and the same map with ablation lesions colored in red (**B**).

LA endocardial voltage mapping was performed in SR before radiofrequency ablation (Figures 3A,B). Endocardial contact during point acquisition was validated by a stable contact signal for >2 beats. All points recorded in SR were analyzed to exclude mechanically induced premature beats. Any area with an abnormal voltage resulting from inadequate contact between the circular catheter and LA tissue was reanalyzed with a 4-mm irrigated contact-force ablation catheter (ThermoCool® SmartTouch®; Biosense Webster, Diamond Bar, CA, USA, or Tacticath®; Abbott, St Paul, MN, USA). The LA was divided into six anatomical regions: posterior; anterior; septal; lateral; LAA; and inferior. The roof was part of the anterior region as previously described (13). The bipolar voltage amplitude was recorded for every point and within each individual region. The median LA and regional bipolar voltage amplitude measurements were calculated. Left atrial intracavitary volume (LAIV) excluding LAA was obtained for each patient after LA anatomic reconstruction and was expressed in milliliters. The left atrial intracavitary volume index (LAIVI) corresponded to the LAIV indexed to the body surface (expressed in ml m^2^). LVZ was defined as sites of >3 adjacent low-voltage points with a bipolar peak-to-peak voltage amplitude of <0.5 mV (14) and covering >5% of the LA surface area (LVZ surface/LA surface >5% without LAA orifice, the pulmonary venous antral region, and mitral valve). This threshold value corresponds to the lowest degree of atrial fibrosis detected using LGE-MRI (15). LVZ extent was categorized as stage I (no or discrete LVZ, ≤5%), II (mild, >5% to ≤20%), III (moderate, >20% to ≤35%), and IV (severe, >35%) according to the UTAH fibrosis classification (15). The surface (cm^2^) of each atrial region and of the LVZ within each atrial region was measured using the 3D-EAM software.

### Catheter ablation procedure

Irrigated radiofrequency energy was applied point by point with a target temperature of 43°C, infusion rate of 17 mL/mn via the irrigated ablation catheter, and maximal power limit of 35 W (30–35 W for the anterior wall and 20–25 W for the posterior wall) using the Biosense Webster ablation index by targeting 450–500 and 350–400 for anterior and posterior segments, respectively. For the Abbott's procedure, the lesion size index (LSI) was used by targeting 5.5 and 4.5 for the anterior and posterior walls, respectively. These same target values were applied to all patients without differentiating between the two groups. PVI was widely encircled in all patients with point-by-point applications using a 4-mm irrigated contact-force ablation catheter (ThermoCool® SmartTouch®; Biosense Webster, Diamond Bar, CA, USA, or Tacticath®; Abbott, St Paul, MN, USA) to create the contiguous lesions. For stage I patients, only circumferential PVI was performed, whereas an additional LVZ-guided substrate ablation was carried out for stage II–IV patients. Linear ablation across LVZs was performed when the LVZ ablation area could be considered a critical isthmus site for potential macro-reentrant tachycardia. Thus, when identifying large LVZs close to the roof or in the anterior LA, linear lesions were created to connect the LVZ ablation area to anatomical obstacles to prevent roof-dependent or peri-mitral atrial flutter. Linear lesions were also performed to isolate large LVZs from the rest of the healthy atrium, such as a posterior box by a roofline and an infero-posterior line. Atrial burst pacing to refractoriness was performed. Inducible atrial tachycardias (AT) were ablated with AT termination without reducibility. No additional ablation was performed in cases of induced AF.

### Patient follow-up

After the catheter procedure, AADs were continued in all patients for at least 3 months to prevent early arrhythmia recurrences. Patients were reviewed by their cardiologist at 3 months and then every 6 months until the 42nd month. At each visit, 12-lead electrocardiogram (ECG) and routine 24-h ECG Holter monitoring were recorded to document the recurrence of atrial arrhythmias (AAs), which was deﬁned as the presence of any documented atrial fibrillation, atrial flutter, and atrial tachycardia lasting over 30 s. The blanking period corresponded to arrhythmic episodes occurring within the ﬁrst 3 months and was not included in the evaluation of ﬁnal success rates. AADs were gradually discontinued at 3–6 months after ablation in the absence of AA recurrence at the physician's discretion.

### Statistical analysis

Categorical variables were presented as absolute values and percentages. A statistical analysis of the categorical variables between the two groups was performed using a chi-square test or Fisher's exact test. The Shapiro–Wilk test was used to determine the Gaussian distribution for each quantitative variable. Continuous variables were expressed as means with standard deviations (SD) for normally distributed data. The variables with a non-normally distribution were presented as medians and interquartile ranges (25th–75th interquartile range). The differences between the continuous variables were analyzed for statistical significance using the Student’s *t*-test or the Wilcoxon test, depending on data distribution.

The relationships between continuous variables were analyzed using Pearson’s correlation coefficients for parametric data. Kaplan–Meier survival curves were used to analyze freedom from AA recurrence after a single AF catheter procedure between obese patients and non-obese patients using the log-rank test with Bonferroni correction. Binominal logistic regression was applied to calculate the odds ratio (OR) and 95% confidence interval (CI) of independent variables associated with AA recurrence. Variables selected for testing in the multivariate analysis were those with *p* < 0.1 in the univariate analysis. All statistical analyses were performed using SPSS statistical software, version 23.0 (IBM Corp.). A two-tailed *p*-value <0.05 was considered statistically significant.

## Results

### Baseline characteristics

Demographic and clinical characteristics of the patients are shown in Table 1. The patients were mainly men (69.8%). Compared to non-obese patients, obese patients were significantly younger [62 (56–69) vs. 66.5 (62–72) years, *p* < 0.01] with more cardiovascular risk factors: hypertension [49 (75.38%) vs. 37 (50%), *p* < 0.01]; diabetes mellitus [20 (30.77%) vs. 6 (8.11%), *p* < 0.01]; obstructive sleep apnea (OSA) [26 (40%) vs. 16 (21.62%), *p* < 0.03]. Left ventricular ejection fraction was normal in most patients. There was no significant difference in drug treatments between the two groups. Paroxysmal AF history was less frequent in the obese group [15 (23.08%) vs. 34 (45.95%), *p* < 0.01]. Indexed to body surface or not, LA intracavitary volume was similar in the two groups [64.7 ml/m^2^ (57.2–73.4) vs. 60.5 ml/m^2^ (51.6–68.6), *p* = 0.07]. At the beginning of the procedure, 23/65 (35.4%) obese patients and 21/77 (28.4%) non-obese patients were in AF (*p* = 0.48).

**Table 1:** Baseline characteristics of the non-obese and obese groups.

	Non-obese group (*n* = 74)	Obese group (*n* = 65)	*p*-value
Age, years	66.5 (62–72)	62 (56–69)	<0.01
Female sex, *n* (%)	20 (27.03%)	22 (33.85%)	0.49
Weight, kg	81.8	102	<0.01
BMI, kg/m^2^	26.7 (24.3–28.1)	32.7 (31.4–37)	<0.01
Dyslipidemia, *n* (%)	25 (33.78%)	25 (38.46%)	0.69
Hypertension, *n* (%)	37 (50%)	49 (75.38%)	<0.01
Diabetes mellitus, *n* (%)	6 (8.11%)	20 (30.77%)	<0.01
Smoking, *n* (%)	11 (14.86%)	10 (15.38%)	1
OSA, *n* (%)	16 (21.62%)	26 (40%)	0.03
Coronary artery disease, *n* (%)	8 (10.81%)	7 (10.77%)	1
Paroxysmal AF history, *n* (%)	34 (45.95%)	15 (23.08%)	<0.01
Time to treatment, days	739 (204–2071)	513 (218–1524)	0.46
Reported AF duration, months			0.73
<3	56 (75.68%)	52 (81.25%)	
≥3 to <6	10 (13.51%)	5 (7.81%)	
≥6 to <9	4 (5.41%)	5 (7.81%)	
≥9 to <12	1 (1.35%)	1 (1.56%)	
≥12	3 (4.05%)	1 (1.56%)	
CHA_2_DS_2_-VASc Score			0.49
0	8 (10.81%)	5 (7.69%)	
1	20 (27.03%)	14 (21.54%)	
2	19 (25.68%)	15 (23.08%)	
3	9 (12.16%)	17 (26.15%)	
≥4	18 (24.32%)	14 (21.55%)	
Beta Blocker	59 (79.73%)	56 (86.15%)	0.44
ACEi/ARB	40 (54.05%)	46 (70.77%)	0.06
Aldosterone receptor antagonist	15 (20.27%)	14 (21.54%)	1
P-wave duration, ms	122.97 ± 23.65	116.65 ± 24.67	0.14
eGFR, ml/min/1.73^2^	78 (69–93)	81 (69–96)	0.71
LVEF, %	61 (50–68)	58 (50–63)	0.16
TTE—LA volume index, ml/m^2^	42 (31–52)	40 (31–51)	0.69
Systolic pulmonary artery pressure, mmHg	30 (26–32)	28 (24–30)	0.17
Per-procedural LAIV excluding LAA, ml	136 (115–150)	129 (110–160)	0.68
Per-procedural LAIVI excluding LAA, ml/m^2^	64.7 (57.2–73.4)	60.5 (51.6–68.6)	0.07
AF at admission before procedure	21 (28.4%)	23 (35.4%)	0.48
Antiarrhythmic drugs prior to procedure *n* (%)	69 (93.4%)	61 (94.1%)	0.92
Amiodarone	59 (80%)	50 (77%)	0.76
Flecainide	9 (12%)	9 (14%)	0.98
Sotalol	1 (1.4%)	2 (3.1%)	0.6

ACEi/ARB, angiotensin-converting enzyme inhibitor/angiotensin II receptor blocker; eGFR, estimated glomerular filtration rate; LVEF, left ventricle ejection fraction; TTE, transthoracic echocardiography.

Data are presented as a value (with percentage) for categorical variables, median (25th–75th percentile) or mean ± SD for quantitative variables. A two-tailed *p*-value < 0.05 was considered significant.

Time to treatment = time from first clinical diagnosis of AF to ablation procedure.

### EAT thickness in non-obese and obese groups

No differences could be seen between the obese and non-obese groups regarding EAT thickness in the left atrial posterior wall (LA-descending thoracic aorta, LA-esophagus, LA-right pulmonary hilum) nor in the anterior, lateral, or inferior walls (Table 2).

**Table 2:** Periatrial fat thickness analysis in non-obese and obese groups.

	Non-obese group (*n* = 74)	Obese group (*n* = 65)	*p-*value
Posterior wall			
LA-descending thoracic aorta, mm	7.9 (6.4–10)	7.3 (5.5–9)	0.11
LA-esophagus, mm	5.1 (3.8–6.6)	4.4 (3.7–5.7)	0.4
LA-right pulmonary hilum, mm	10.3 ± 2.4	10.7 ± 1.8	0.36
Anterior wall			
LA-ascending thoracic aorta, mm	8.4 (6.7–9.6)	8.9 (6.3–10.5)	0.39
LA-pulmonary artery, mm	10.2 (8.2–12.4)	10.3 (8.5–12.9)	0.56
Lateral wall			
LA-mitral isthmus, mm	6.9 (5.5–8.7)	7.4 (6.1–10.3)	0.08
LA-appendage, mm	8.9 (7.6–10.4)	8.9 (7.7–11.9)	0.5
LA-floor, mm	6.7 (5.3–8.5)	7.5 (5.9–8.8)	0.12

All data are presented as median (25th–75th percentile) or mean ± SD. A two-tailed *p-*value < 0.05 was considered significant.

### Bipolar voltage assessment in non-obese and obese groups

Of the procedures, 73 (52.5%) were performed with the Afocus II. The 66 (47.5%) remaining procedures were performed with the Lasso™ NAV. No difference was found in the use of the type of mapping catheter between the obese and non-obese groups [Abbott: 36 (55%) vs. 37 (50%), *p* = 0.64 and Biosense Webster: 29 (45%) vs. 37 (50%), *p* = 0.64].

The number of mapping points per patient was similar in the two groups [998 (673–1,662) vs. 1,031 (566–2,003), *p* = 0.83] (Table 3). The global LA bipolar voltage was not significantly different in the obese group compared to the non-obese group [2.2 (1.4–2.6) mV vs. 1.8 (1.2–2.7) mV, *p* = 0.29] (Table 3). When comparing LA bipolar voltage amplitudes according to atrial regions, there was also no difference in bipolar voltage amplitude in the anterior [1.7 (1.1–2.6) mV vs. 1.6 (1–2.4) mV, *p* = 0.25], septal [1.6 (1–2.3) mV vs. 1.4 (0.8–2.2) mV, *p* = 0.29], posterior [2.3 (1.3–3.2) mV vs. 1.9 (1.1–2.8) mV, *p* = 0.22], inferior [2 (1.2–2.7 mV) vs. 1.8 (1.1–2.6 mV), *p* = 0.34], or lateral [2.3 (1.3–3.4 mV) vs. 2.1 (1.2–2.8 mV), *p* = 0.29] regions nor the LAA [2.9 (1.8–3.9 mV) vs. 2.6 (1.7–3.5, mV *p* = 0.13] between the obese and non-obese patients.

**Table 3:** Median regional distribution of bipolar voltage amplitudes of the non-obese and obese groups.

	Non-obese group (*n* = 74)	Obese group (*n* = 65)	*p-*value
Median total mapping points/per patient	998 (673–1662)	1,031 (566–2003)	0.83
Median and regional bipolar voltage amplitude, mV			
Global left atrium	1.8 (1.2–2.7)	2.2 (1.4–2.6)	0.29
Anterior	1.6 (1–2.4)	1.7 (1.1–2.6)	0.25
Septal	1.4 (0.8–2.2)	1.6 (1–2.3)	0.29
Posterior	1.9 (1.1–2.8)	2.3 (1.3–3.2)	0.22
Inferior	1.8 (1.1–2.6)	2 (1.2–2.7)	0.34
Lateral	2.1 (1.2–2.8)	2.3 (1.3–3.4)	0.29
Left atrial appendage	2.6 (1.7–3.5)	2.9 (1.8–3.9)	0.13

Data are presented as median (25th–75th percentile). A two-tailed p-value < 0.05 was considered significant.

### Low-voltage zone assessment in non-obese and obese groups

LVZs were observed in 27.3% of patients in the whole cohort. LVZs were less frequent in the obese group compared to the non-obese group [12% (18.8%) vs. 26% (35.1%), *p* = 0.05] (Table 4).

**Table 4:** LVZs extent and distribution analysis in non-obese and obese groups.

	Non-obese group (*n* = 74)	Obese group (*n* = 65)	*p-*value
LVZ extent calculated as the percentage of LA surface area			
LVZ, *n* (%)	26 (35.1%)	12 (18.8%)	0.05
Mild LVZ, *n* (%)	11 (14.9%)	5 (7.8%)	0.31
Moderate LVZ, *n* (%)	7 (9.5%)	5 (7.8%)	0.97
Severe LVZ, *n* (%)	8 (10.8%)	2 (3.1%)	0.11
No LVZ, *n* (%)	48 (64.9%)	52 (81.3%)	0.05
Number of regional LVZ, *n* (%)			
Anterior	28 (37.8%)	13 (20.3%)	0.04
Septal	22 (29.7%)	14 (21.9%)	0.39
Posterior	14 (18.9%)	6 (9.4%)	0.18
Inferior	6 (8.1%)	2 (3.1%)	0.29
Lateral	2 (2.7%)	2 (3.1%)	1
LAA	4 (5.4%)	2 (3.1%)	0.69
LVZ surface (cm^2^) indexed to regional atrial surface (cm^2^), %			
LVZ/LA area	14 (5.5–33)	8.2 (3.4–22.6)	0.17
Anterior LVZ/anterior area	29.8 (13.9–52.3)	18.3 (9.2–32)	0.29
Septal LVZ/septal area	18.9 (14–41.4)	11.4 (8.4–33.1)	0.18
Posterior LVZ/posterior area	28.7 (11.9–42.3)	35.9 (19.3–44.3)	0.51
Inferior LVZ/inferior area	12.3 (7.7–27.2)	9.8 (6.2–14.5)	0.84
Lateral LVZ/lateral area	26.4 (15.6–68.9)	10.2 (9.3–35.7)	0.74
LAA LVZ/LAA area	16.1 (9.7–23.7)	11 (9.7–14)	0.79

All data are presented as a value (percentage) for categorical variables or median (25th–75th percentile) for quantitative variables. A two-tailed p-value < 0.05 was considered significant.

When analyzing LVZs according to the atrial region, anterior LVZs were less frequently seen in the obese group [13% (20.3%) vs. 28% (37.8%), *p* = 0.04]. In the other atrial regions, no significant difference in LVZs could be observed between the two groups. LA surface indexed-LVZ extents were not significantly different between the two groups [8.2% (3.4%–22.6%) vs. 14% (5.5%–33%), *p* = 0.17]. LA surface indexed-LVZ extents according to regional atrial surface were also not significantly different, even if the obese group presented less extent, particularly in the anterior, septal, inferior, and lateral regions [18.3% (9.2%–32%) vs. 29.8% (13.9%–52.3%), *p* = 0.29; 11.4% (8.4%–33.1%) vs. 18.9% (14%–41.4%), *p* = 0.18; 9.8% (6.2%–14.5%) vs. 12.3% (7.7%–27.2%), *p* = 0.84; 10.2% (9.3%–35.7%) vs. 26.4% (15.6%–68.9%), *p* = 0.74, respectively].

### Association between EAT thickness and regional LVZ extent

To analyze the relationship between EAT thickness and LVZ extent indexed to regional atrial surface, the Pearson correlation coefficients were calculated, as shown in Table 5. No significant correlation was found in non-obese patients. In obese patients, the EAT thickness of LA-esophagus and the indexed posterior LVZ extent showed a positive Pearson correlation coefficient of 0.45 (*p* = 0.0001) (Table 5). LA-mitral isthmus was also modestly correlated to indexed lateral LVZ extent (Pearson coefficient 0.26, *p* = 0.04). There was no significant correlation between LVZ extent and EAT in the other regions.

**Table 5:** Correlation between periatrial fat thicknesses values and percentage of regional LVZ extent in the non-obese patients (A) and in the obese patients (B).

	*r*	*p*-value	95% CI
(A)			
Posterior LVZ/posterior area (%)			
LA-esophagus	0.000	1.000	−0.288 to 0.263
LA-descending thoracic aorta	0.138	0.262	−0.087 to 0.351
LA-right pulmonary hilum	−0.173	0.140	−0.345 to −0.005
Anterior LVZ/anterior area (%)			
LA-ascending thoracic aorta	−0.056	0.633	−0.286 to 0.157
LA-pulmonary artery	−0.077	0.514	−0.259 to 0.105
Lateral LVZ/lateral area (%)			
LA-mitral isthmus	0.129	0.277	−0.149 to 0.407
LAA LVZ/LAA area (%)			
LA- appendage	−0.033	0.780	−0.137 to 0.098
Inferior LVZ/inferior area (%)			
LA-floor	0.160	0.172	−0.070 to 0.378
(B)			
Posterior LVZ/posterior area (%)			
LA-esophagus	0.450	0.0001	−0.093 to 0.735
LA-descending thoracic aorta	−0.023	0.860	−0.161 to 0.104
LA-right pulmonary hilum	−0.046	0.720	−0.226 to 0.222
Anterior LVZ/anterior area (%)			
LA-ascending thoracic aorta	0.040	0.757	−0.321 to 0.382
LA-pulmonary artery	0.099	0.441	−0.158 to 0.375
Lateral LVZ/lateral area (%)			
LA-mitral isthmus	0.260	0.040	−0.082 to 0.555
LAA LVZ/LAA area (%)			
LA-appendage	−0.057	0.660	−0.229 to 0.194
Inferior LVZ/inferior area (%)			
LA-floor	−0.057	0.656	−0.250 to 0.486

*r*, Pearson’s correlation coefficient.

Significant value (*p* < 0.05).

### Ablation procedure

All PVs were successfully isolated during CA. PVI alone was performed in 88 (63.3%) patients of the overall cohort, while the remaining 51 (36.7%) patients had an additional LVZ-guided substrate ablation. Compared to non-obese patients, PVI alone was more frequently performed in obese patients [47 (72.3%) vs. 41 (55.4%), *p* = 0.06] but the difference was not significant. CTI ablation before or during the procedure was performed in the same proportion in the two groups [13 (17.6%) vs. 13 (20%), *p* = 0.88]. Total RF duration and fluoroscopy time were also similar (31.9 ± 10.5 min vs. 32.1 ± 11.8 min, *p* = 0.76; 25.1 ± 11.2 min vs. 24.9 ± 9.4 min, *p* = 0.66) (Supplementary Table S1).

### Long-term clinical outcome after an AF ablation procedure

**Figure 4:**
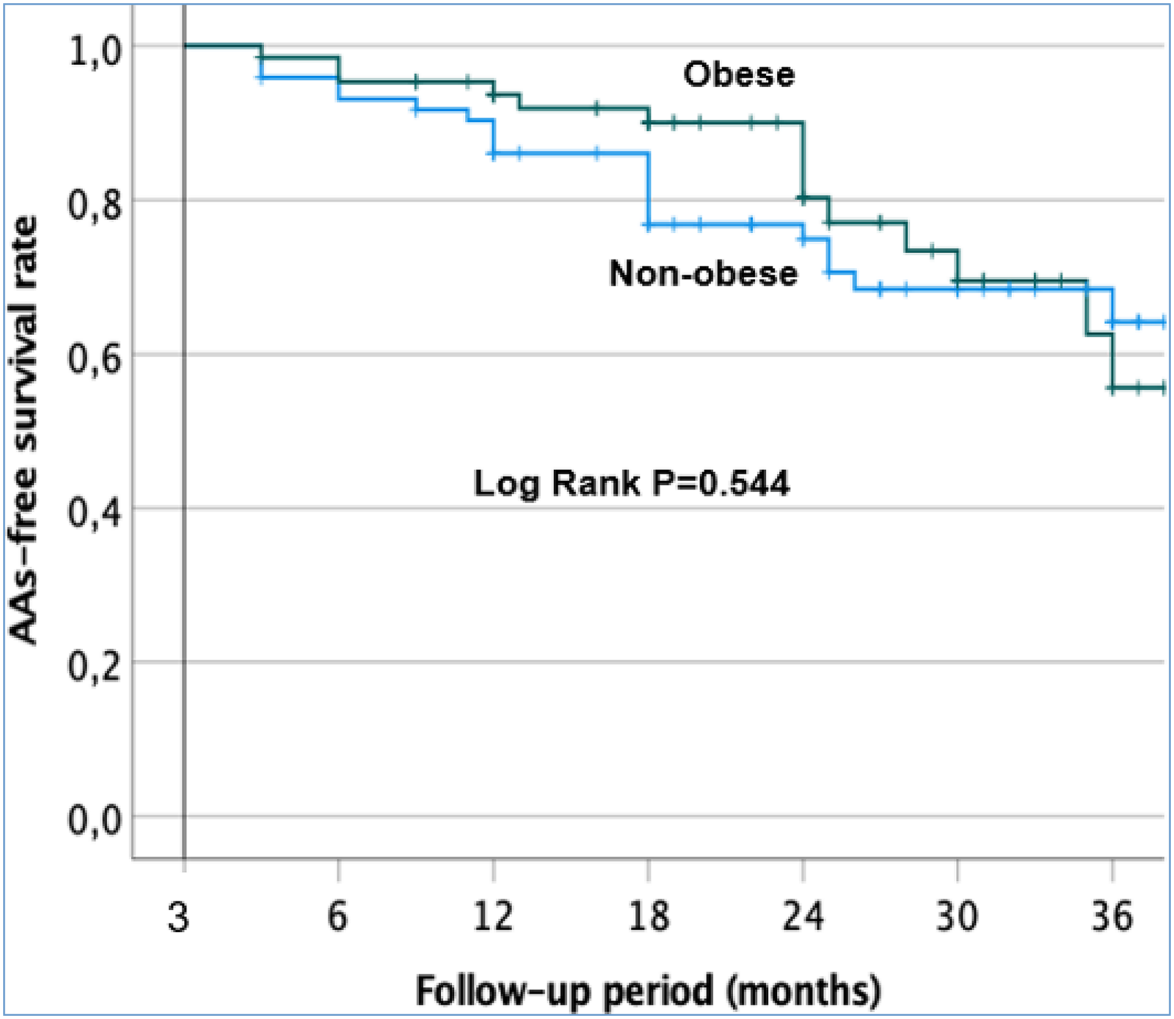
Kaplan–Meier survival curves showing the cumulative AAS recurrence-free survival rates in the obese and non-obese patients after a single procedure.

After a follow-up of 38.1 months (range 34.8–41.4), AAs recurred in 37/138 (26.8%) of the overall cohort after CA. The Kaplan–Meier survival curves are shown in Figure 4. There was no significant difference in AA-free survival rate after CA in the obese and non-obese groups (Log-rank test, *p* = 0.544). The percentage of patients remaining free from AF/AT 12 months after CA was 93% ± 3% in the obese group and 86% ± 4% in the non-obese group (Figure 4). At 24 months after ablation, 80% ± 6% of the obese group and 75% ± 5% of the non-obese group remained free from AF/AT. Finally, 77% ± 6% of the obese group and 64% ± 7% of the non-obese group remained free from AF/AT 36 months after CA.

Among patients without AA recurrence at 12 months, AADs were discontinued in 74.4% of the whole cohort: 68.3% in the obese group and 80% in the non-obese group (*p* = 0.2).

Finally, we found no differences in AA-free survival rate after one procedure between obese patients who received PVI alone and those with additional LVZ ablation (log-rank test, *p* = 0.712). The percentage of obese patients remaining free of AF/AT after 24 months was 77% ± 8% of those who received PVI alone and 76% ± 13% of those who received additional LVZ ablation (Supplementary Figure S1). For non-obese patients, no differences in AA-free survival rate were observed between PVI alone and additional LVZ ablation (log-rank test, *p* = 0.548). The percentage of non-obese patients remaining free of AF/AT after 24 months was 68% ± 8% of those who received PVI alone and 75% ± 9% of those who received additional LVZ ablation (Supplementary Figure S2).

During follow-up, we did not observe differences in body weight [102 (94–112) kg vs. 100 (91–112) kg, *p* = 0.15] and BMI [33 (31–37) kg/m^2^ vs. 32 (31–36) kg/m^2^, *p* = 0.2] in the obese group before and 1 year after AF ablation. An initial weight loss of ≥10% resulted in only 7.7% of obese patients with persistent AF. Of these obese patients, 30% had even gained excess weight at the 1-year follow-up.

### Predictive factors of AA recurrence after CA

To evaluate the predictors of AA recurrence after a single CA procedure, univariate and multivariate analyses were carried out in both the obese and the whole cohorts.

In the obese population, P-wave duration (PWD), LA indexed intracavitary volume, and LA-mitral isthmus were chosen as variables (*p* < 0.1) for a multivariate analysis, but none of them was a significant predictor of AF recurrence (Table 6A).

**Table 6:** Univariate and multivariate analyses for prediction of AAs recurrence after catheter ablation in the obese cohort (A) and in the whole cohort (B).

Variable	Univariate analysis			Multivariate analysis		
	OR	95% CI	*p-*value	OR	95% CI	*p-*value
(A)						
Age	0.98	0.877–1.104	0.78	—	—	—
AF duration > 6 months	0.34	0.030–3.802	0.38	—	—	—
Time to treatment	1	1.000–1.001	0.35	—	—	—
BMI	0.76	0.467–1.240	0.27	—	—	—
Hypertension	3.62	0.464–28,195	0.22	—	—	—
OSA	0.21	0.020–2.113	0.18	—	—	—
CHA_2_DS_2_-VASc score ≥2	0.84	0.082–8.681	0.89	—	—	—
eGFR	1.02	0.961–1.086	0.49	—	—	—
LAIVI	1.06	1.008–1.123	0.03	—	—	0.991
ACEI/ARB	2.75	0.357–21,187	0.33	—	—	—
P-wave duration	1.07	1.005–1.141	0.04	—	—	0.980
LA-esophagus	1.22	0.782–1.899	0.38	—	—	—
LA-descending thoracic aorta	1.18	0.905–1.525	0.23	—	—	—
LA-right pulmonary hilum	0.99	0.559–1.759	0.98	—	—	—
LA-ascending thoracic aorta	1.03	0.715–1.475	0.89	—	—	—
LA-pulmonary artery	0.95	0.699–1.294	0.75	—	—	—
LA-mitral isthmus	1.34	0.998–1.800	0.05	—	—	0.995
LA-left atrial appendage	0.97	0.715–1.322	0.86	—	—	—
LA-floor	1.26	0.976–1.619	0.08	—	—	—
LA bipolar voltage	0.72	0.237–2.215	0.57	—	—	—
Presence of LVZs	0.69	0.065–7.253	0.76	—	—	—
Only PVI	2.75	0.357–21.187	0.33	—	—	—
(B)						
Female gender	2.59	0.548–12.229	0.23	—	—	—
Age	1	0.939–1.067	0.98	—	—	—
Time to treatment	1	1.000–1.001	0.56	—	—	—
BMI	0.94	0.840–1.059	0.32	—	—	—
Dyslipidemia	1.31	0.382–4.494	0.67	—	—	—
Hypertension	2.07	0.657–6.550	0.214	—	—	—
OSA	0.33	0.105–1.061	0.06	0.62	0.233–1.635	0.33
Paroxysmal AF history	1.27	0.369–4.344	0.71	—	—	—
CHA_2_DS_2_-VASc score ≥2	1.28	0.394–4.159	0.68	—	—	—
eGFR	1.01	0.976–1.045	0.58	—	—	—
LAIVI	1.02	0.992–1.057	0.15	—	—	—
ACEI/ARB	1.04	0.320–3.357	0.95	—	—	—
Aldosterone receptor antagonist	0.26	0.081–0.857	0.03	0.78	0.258–2.352	0.66
P-wave duration	1.045	1.014–1.077	0.004	1.03	1.003–1.050	0.03
LA-esophagus	1.29	0.991–1.668	0.06	1.13	0.884–1.444	0.33
LA-descending thoracic aorta	1.14	0.968–1.330	0.12	—	—	—
LA-right pulmonary hilum	0.96	0.732–1.251	0.75	—	—	—
LA-ascending thoracic aorta	1.05	0.847–1.301	0.66	—	—	—
LA-pulmonary artery	1.11	0.935–1.305	0.24	—	—	—
LA-mitral isthmus	1.28	1.052–1.562	0.01	1.21	1.013–1.433	0.04
LA-left atrial appendage	0.9	0.724–1.119	0.34	—	—	—
LA-floor	1.1	0.930–1.307	0.26	—	—	—
LA bipolar voltage	0.97	0.533–1.758	0.92	—	—	—
Presence of LVZ	0.85	0.245–2.944	0.8	—	—	—
Only PVI	1.07	0.331–3.476	0.91	—	—	—

eGFR, estimated glomerular filtration rate; ACEI/ARB, angiotensin-converting enzyme inhibitor/angiotensin receptor blocker.

Data are presented as an odds ratio with 95% CI. A two-tailed *p*-value < 0.05 was considered significant.

Time to treatment = time from first clinical diagnosis of AF to ablation procedure.

In the whole cohort, obstructive sleep apnea, treatment by aldosterone receptor antagonist, PWD, EAT LA-esophagus and EAT LA-mitral isthmus were selected as variables (*p* < 0.10) for a multivariate analysis. PWD (OR1.03, 95% CI 1–1.05, *p* = 0.03) and EAT LA-mitral isthmus (OR 1.21, 95% CI 1–1.4, *p* = 0.04) were significant predictors of AF recurrence after CA (Table 6B).

## Discussion

As shown in previous studies (16, 17), we reported an increased occurrence of arterial hypertension, diabetes, and obstructive sleep apnea in the obese cohort, all known to be predisposing factors for AF. We also observed fewer cases with a history of paroxysmal AF in the obese group compared to the non-obese group. These data are consistent with other studies (16, 18) observing that AF became more persistent and less paroxysmal with increasing BMI. The underlying mechanisms are not fully understood and are probably multifactorial, suggesting that cardiovascular risk factors may promote the rapid progression from paroxysmal to persistent AF in obese patients through LA electrical remodeling or left ventricular diastolic dysfunction.

EAT has gained attention in the last few years and could be the link between obesity and the onset of AF. Thanassoulis et al. showed that the extent of epicardial fat was associated with AF occurrence whereas intrathoracic and abdominal fat were not (6). Batal et al. also evidenced that posterior EAT was associated with AF burden (8).

In addition, Wong et al. observed that patients with extensive EAT had early AF recurrence after the ablation procedure (7). Finally, a meta-analysis also underlined an association between increased EAT and AF recurrence in patients undergoing AF ablation (19). As evidenced in previous studies, EAT measured on the lateral wall was also an independent predictor of AF recurrence in our study.

Even if obesity is closely associated with AF occurrence and persistent AF, BMI might not be directly linked to EAT extent. Moreover, the amount of fat is probably not the best way of evaluating EAT. As demonstrated by Venteclef et al., EAT is a reservoir of adipokines acting as pro-inflammatory actors (9). Its metabolic activity measurement could be more relevant but more difficult to highlight. Wang et al. showed that the increased inflammatory activity of LA-EAT was positively correlated with AF (20). In addition, Ciuffo et al. found that the quality of the left periatrial adipose tissue, assessed by CT attenuation, was an independent predictor of AF recurrence after ablation (21).

The quality of LA-EAT, rather than its quantity, may also play an important role in the gender disparity of LA remodeling between LA-EAT and LA-LVZ. All these findings remain to be confirmed and prompt further investigation in future studies.

Matos et al. recently showed for the first time a weak correlation between EAT measured with computed tomography (CT) and LA fibrosis measured with cardiac magnetic resonance. EAT was more strongly associated with AF recurrence than LA fibrosis. This finding suggested that LA fibrosis is not the main mechanism linking EAT and AF (22).

EAT might play a role in AF onset and progression through other mechanisms, such as shortening the potential duration of action (23). In addition, large amounts of ganglionated plexi and cholinergic and adrenergic nerves are mostly embedded in the EAT. An autonomic nervous system (ANS) dysfunction could change the endocrine properties of the epicardial fat through a secretory activity enhancement and adipogenesis (24).

EAT could play an important role in cardiomyocyte function by channel dysfunctions (25) and in atrial inflammation via interleukin-6 elevation (26) inducing atrial electrical remodeling by downregulating cardiac connexins. Recently, NLRP3-inflammasome has been demonstrated to be a key driver of obesity-induced AF (27).

These mechanisms could play an important role in the rapid progression from paroxysmal to persistent AF in obese patients. Moreover, time to treatment was also shorter in the obese cohort, probably because of the persistent behavior of AF. All these elements listed above could explain both the younger age of this cohort and the absence of severe LA dilatation with thus less LVZ in obese group. Indeed, today we know that the presence of LVZ was associated both with age (13, 28) and LA enlargement (13, 29).

Overall, obesity-induced FA could be initiated by EAT-mediated reversible electrophysiological remodeling via electrical and molecular mechanisms as in our younger obese patients.

Indeed, Middeldorp et al. demonstrated that weight loss and management of risk factors may reverse the natural progression of AF. A weight loss of ≥10% resulted in 88% of patients with persistent AF reversal to paroxysmal or no AF, with 45% of patients no longer requiring ablation (30).

In addition, Mahajan et al. demonstrated that weight loss in an obese ovine model was associated with structural and electrophysiological reverse remodeling (31).

Moreover, Schram-Serban et al. recently demonstrated that the percentages of low-voltage potentials were similar between obese and non-obese patients within the majority of atrial locations (posterior LA, inferior LA, mitral isthmus, LAA, right atrium) except the Bachmann bundle using high-resolution epicardial mapping during SR. This mapping was performed during open chest cardiac surgery after sternotomy before connecting the patient to cardiopulmonary bypass circulation (32). These findings are consistent with our data except for the age of the cohort being the same in their study.

Interestingly, BMI was not a predictive factor of AF recurrence after CA. If obesity is clearly identified as a risk factor of AF onset, the impact of BMI on AF ablation outcome is still under debate with contrasting results (16, 33, 34).

Despite fewer LVZs in the obese group, we observed that the EAT amounts on the posterior and lateral walls were significantly correlated with posterior and lateral LVZ extent. The LA surface indexed-LVZ extent was the only one to be increased on the posterior wall compared to the non-obese group, although this was not significant.

Previous studies demonstrated that the presence of EAT was associated with lower bipolar voltage and atrial electrogram fractionation (12, 35). Mahajan et al. even showed that EAT infiltrated the posterior LA in the obese group with reduced endocardial voltage and conduction abnormalities in this region (12). All EAT measurements in their study were consistently greater in the obese group. We could not see such differences in our cohort. When analyzing their population, there were only 16 obese patients and 10 non-obese patients. In the non-obese group, 80% of patients displayed paroxysmal AF, known to be associated with less LA remodeling. Obesity was also defined as a BMI > 27 kg/m^2^. These elements may explain such differences between the two groups. In our cohort, EAT measurements were not significantly different among obese and non-obese patients, explaining the moderate substrate remodeling in our obese group. We could therefore postulate that the EAT-mediated fibrotic irreversible remodeling may occur later with increasing age in obese patients.

Several studies showed that obesity had a negative impact on AF ablation outcomes (17, 33). In our study, we could not find any difference after one single voltage-guided ablation, which had never been evaluated in an obese cohort. A recent study showed that obesity does not adversely affect the outcome of ablation until BMI exceeds 35 kg/m^2^ (17). Only patients with morbid obesity (BMI ≥ 35 kg/m^2^) have a higher recurrence rate of atrial arrhythmias during follow-up in persistent AF compared to other groups. In our obese patients, the median BMI was 32.7 kg/m^2^, which could explain the absence of difference in the outcome after CA.

Sivasambu et al. evidenced that persistent AF ablation outcome in patients undergoing PVI alone with cryoballoon was not affected by BMI. Freedom from AF recurrence at 1 year was around 45%–50% after PVI alone (36). We reported a better outcome in the obese group after a single LVZ-guided ablation in addition to PVI with 68% of patients off drugs at 1 year. Our study is among the first to evaluate the outcome of LVZ-guided ablation in obese persistent AF patients.

The sole predictive factor of recurrence after CA in our cohort was PWD. Several studies have also reported this association (37): a prolonged PWD attesting to an intra- and inter-atrial abnormal conduction as well the duration and direction of atrial depolarization. Dąbrowska-Kugacka et al. also observed that PWD was highly correlated with atrial electroanatomical delay assessed by tissue Doppler echocardiography (38). Some authors reported that a prolonged PWD was correlated with left atrial enlargement (39). A significant association between LVZ and prolonged PWD was also seen in patients with both paroxysmal and persistent AF (40, 41).

Whatever the chosen strategy of persistent AF ablation, electrophysiologists should not be discouraged from proposing an early persistent AF ablation in obese patients, which is associated with weight loss and physical activity, especially if the BMI is <35 kg/m^2^.

### Study limitations

This was a single-center observational study with a limited number of patients. During 3D mapping, although a great number of points were collected, high-density catheters could have been used for a more rapid and better resolution, particularly for LVZ assessment. The study started using a circular catheter. To have homogeneous data, the same type of catheter was used for the whole study. In addition, the discontinuation of AADs could not be obtained in the whole cohort because the follow-up was conducted by the patients' cardiologists, who were probably less motivated to stop AADs in obese patients. Further studies are needed to assess the long-term follow-up after AF ablation in obese patients.

## Conclusion

Obese patients with persistent AF display less electrophysiological substrate remodeling with similar LA volumes, bipolar voltage, and a similar amount of EAT compared to non-obese patients. In obese patients, EAT measured on the posterior and lateral LA wall were correlated with posterior and lateral LVZ extents. Unlike BMI, the EAT extent on the lateral wall and PWD were independent predictors of AF recurrence. Obese patients have a similar and favorable 36-month outcome after one single voltage-guided substrate persistent AF ablation. An early strategy of rhythm control using AF ablation should be proposed in obese patients to improve the cardiovascular prognosis. At the same time, weight loss and management of risk factors are absolutely necessary to reverse the natural progression of AF to avoid EAT-mediated fibrotic irreversible remodeling.

## Data Availability

The original contributions presented in the study are included in the article/**Supplementary Material**, further inquiries can be directed to the corresponding author.
