# Supplementary material for: Left atrial remodeling and voltage-guided ablation outcome in obese patients with persistent atrial fibrillation
Source: Front Cardiovasc Med. 2024 Apr 2;11:1362903. doi: 10.3389/fcvm.2024.1362903 (PMC11018888; doi:10.3389/fcvm.2024.1362903)
Supplement: Supplementary file 1 [file Table1.doc]

**Supplemental Table 1. Procedural data for the non-obese and obese groups**

|  | **Non-obese group (n=74)** | **Obese group (n=65)** | **P value** |
| --- | --- | --- | --- |
| fluoroscopic time, min | 25.1 ± 11.2 | 24.9 ± 9.4 | 0.66 |
| Median total mapping points per patient | 998 (673-1662) | 1031 (566-2003) | 0.83 |
| Total RF duration, min | 31.9 ± 10.5 | 32.1 ± 11.8 | 0.76 |
| AF at admission before procedure | 21 (28.4%) | 23 (35.4%) | 0.48 |
| Only PVI | 41 (55.4%) | 47 (72.3%) | 0.06 |
| CTI ablation before or during procedure | 13 (17.6%) | 13 (20%) | 0.88 |

Data are presented as a value (with percentage) for categorical variables, median (25^th^-75^th^ percentile) or mean±SD for quantitative variables. A two-tailed p value<0.05 was considered significant. Min, minute; *AF, atrial fibrillation; RF= radiofrequency; PVI, pulmonary vein isolation; CTI, cavo-tricuspid isthmus ablation.*
