# Supplementary material for: Left atrial remodeling and voltage-guided ablation outcome in obese patients with persistent atrial fibrillation
Source: Front Cardiovasc Med. 2024 Apr 2;11:1362903. doi: 10.3389/fcvm.2024.1362903 (PMC11018888; doi:10.3389/fcvm.2024.1362903)
Supplement: Supplementary file 2 [file Presentation1.ppt]

## Slide 1
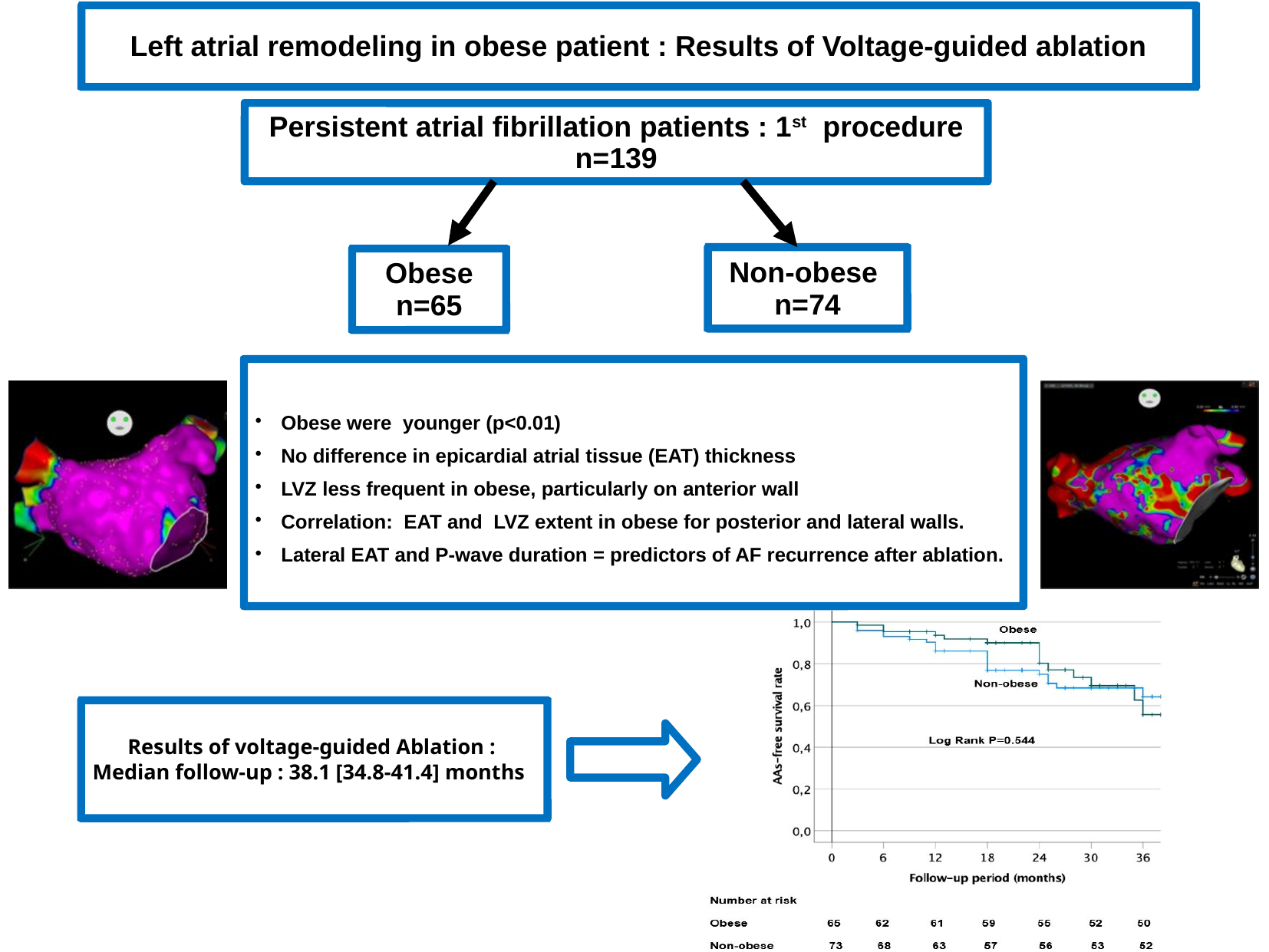

Left atrial remodeling in obese patient : Results of Voltage-guided ablation
Persistent atrial fibrillation patients : 1st procedure
n=139
Non-obese
n=74
Obese
n=65
Obese were younger (p<0.01)
No difference in epicardial atrial tissue (EAT) thickness
LVZ less frequent in obese, particularly on anterior wall
Correlation: EAT and LVZ extent in obese for posterior and lateral walls.
Lateral EAT and P-wave duration = predictors of AF recurrence after ablation.
Results of voltage-guided Ablation :
Median follow-up : 38.1 [34.8-41.4] months
