# Supplementary material for: Left atrial remodeling and voltage-guided ablation outcome in obese patients with persistent atrial fibrillation
Source: Front Cardiovasc Med. 2024 Apr 2;11:1362903. doi: 10.3389/fcvm.2024.1362903 (PMC11018888; doi:10.3389/fcvm.2024.1362903)
Supplement: Supplementary file 3 [file Datasheet1.doc]

**
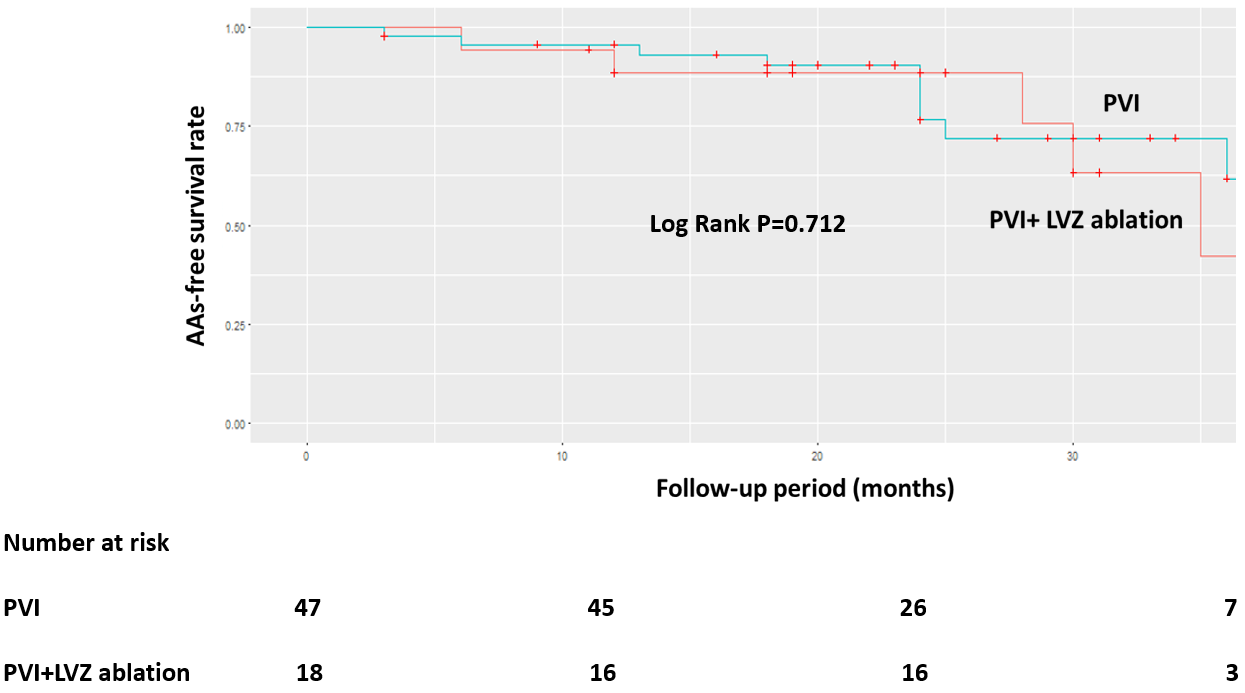
**

**Figure S1.** Kaplan-Meier survival curves showing the cumulative AAs recurrence-free survival rates between obese patients with additional LVZ ablation and those with PVI alone after a single procedure.

**Abbreviations :** *AAs, atrial arrhythmias ; PVI, pulmonary vein isolation ; LVZ, low-voltage zone.*

**
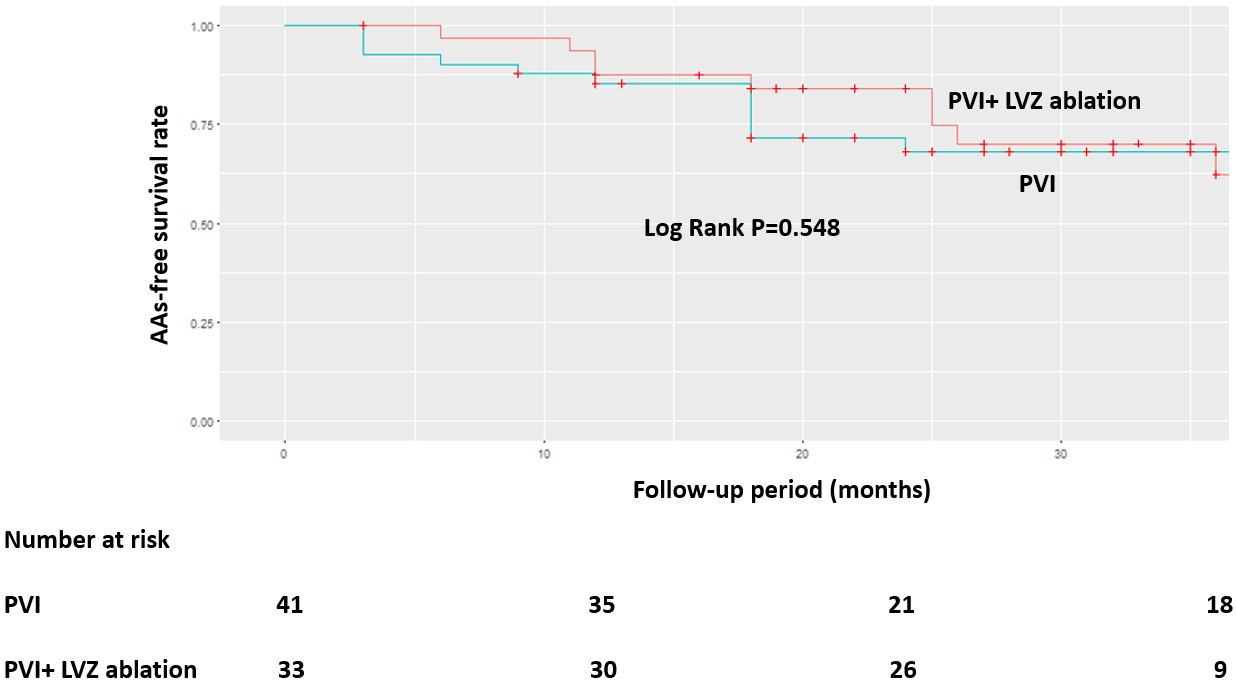
**

**Figure S2.** Kaplan-Meier survival curves showing the cumulative AAs recurrence-free survival rates between non obese patients with additional LVZ ablation and those with PVI alone after a single procedure.

**Abbreviations :** *AAs, atrial arrhythmias ; PVI, pulmonary vein isolation ; LVZ, low-voltage zone.*
